# Supplementary figures and images for: Serum uric acid level and all-cause and cardiovascular mortality in peritoneal dialysis patients: A systematic review and dose-response meta-analysis of cohort studies
Source: PLoS One. 2022 Feb 22;17(2):e0264340. doi: 10.1371/journal.pone.0264340 (PMC8863225; doi:10.1371/journal.pone.0264340)

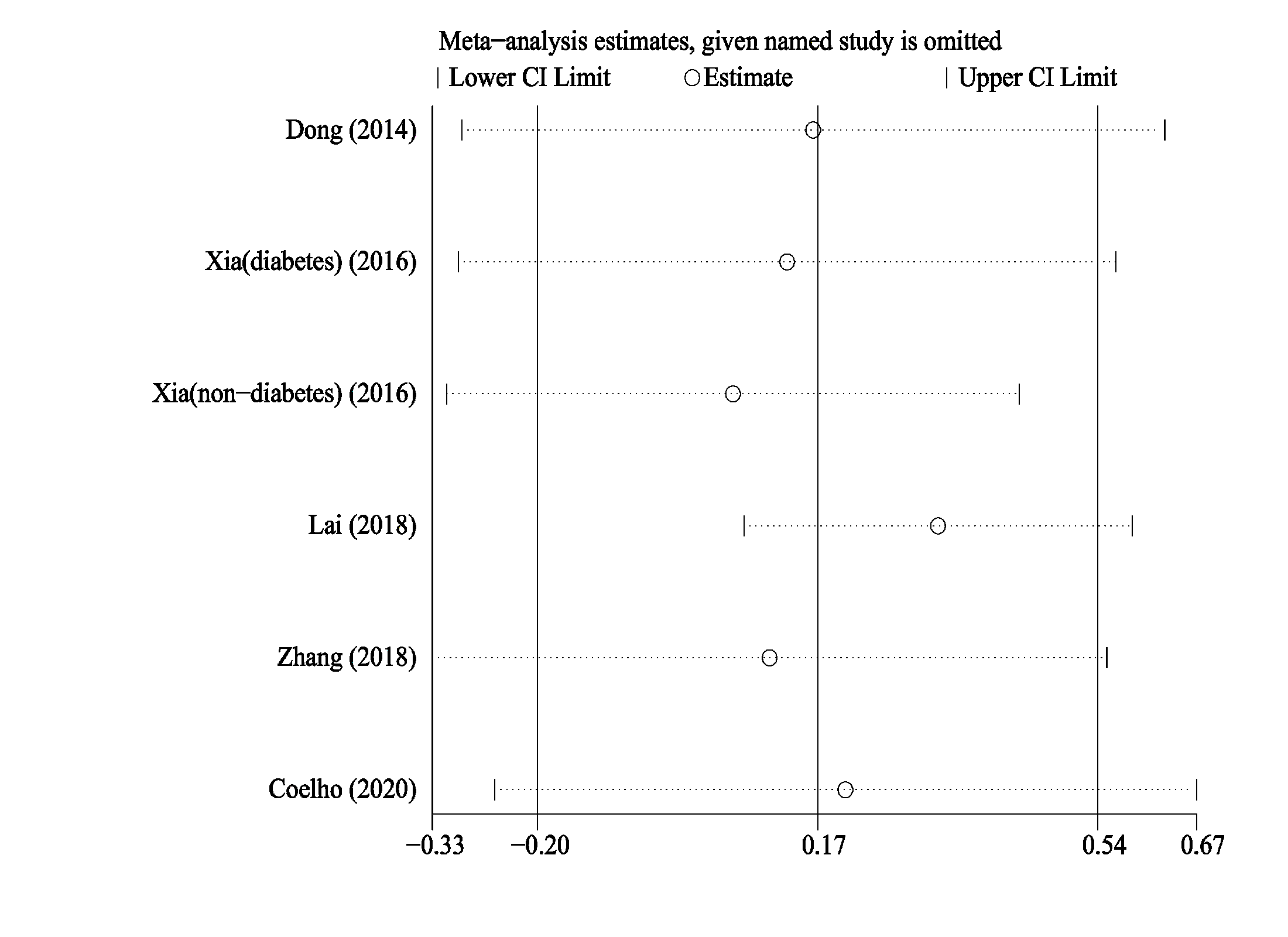

Supplement: S1 Fig — For relationship between SUA by categories (the highest SUA category vs the lowest) and all-cause mortality in PD patients before recalculated the HRs and 95% CIs. HR, hazard ratio; CI, confidence interval. (TIF) [file pone.0264340.s002.tif]

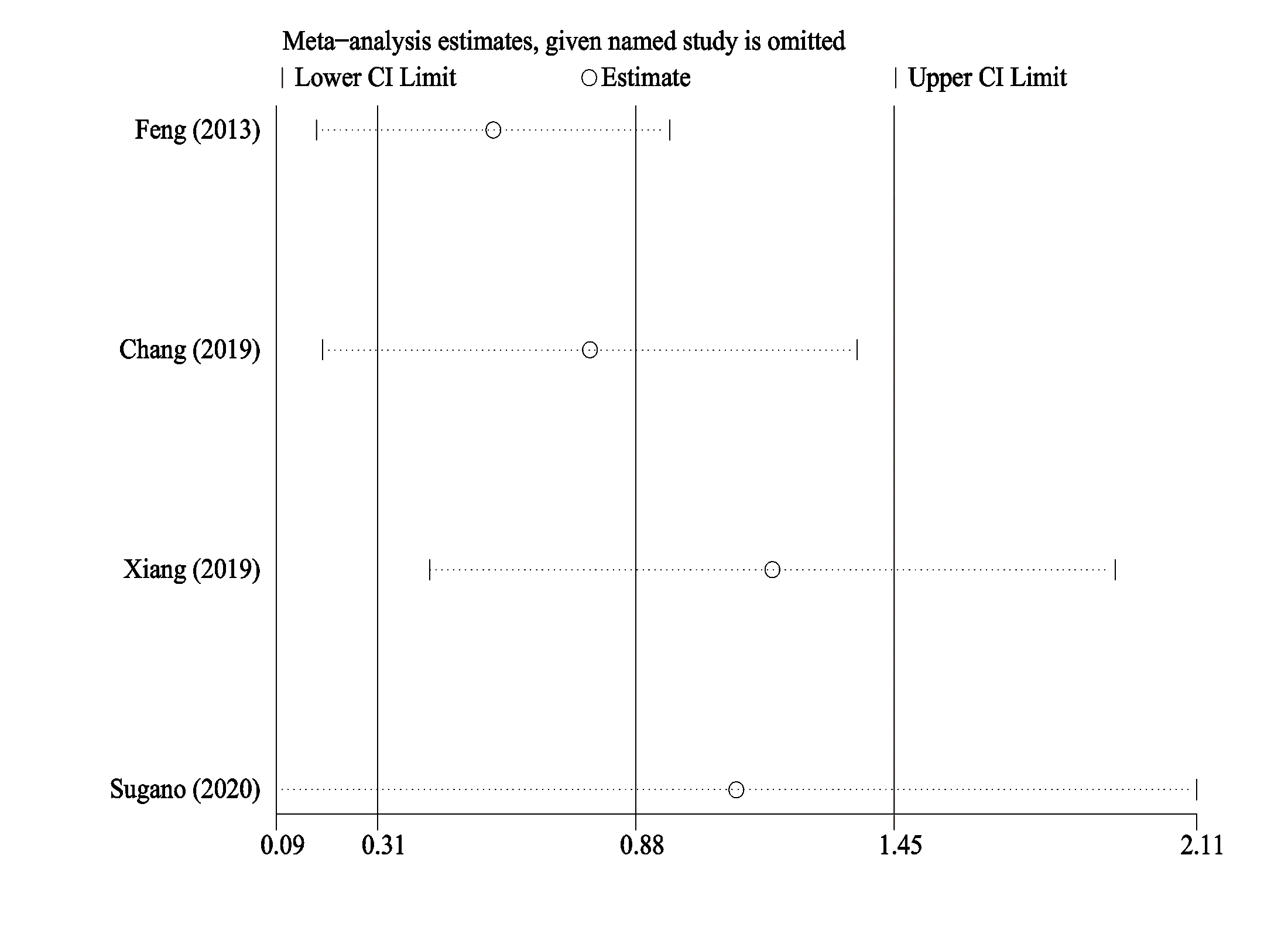

Supplement: S2 Fig — For relationship between SUA by categories (the highest SUA category vs the median) and all-cause mortality in PD patients before recalculated the HRs and 95% CIs. HR, hazard ratio; CI, confidence interval. (TIF) [file pone.0264340.s003.tif]

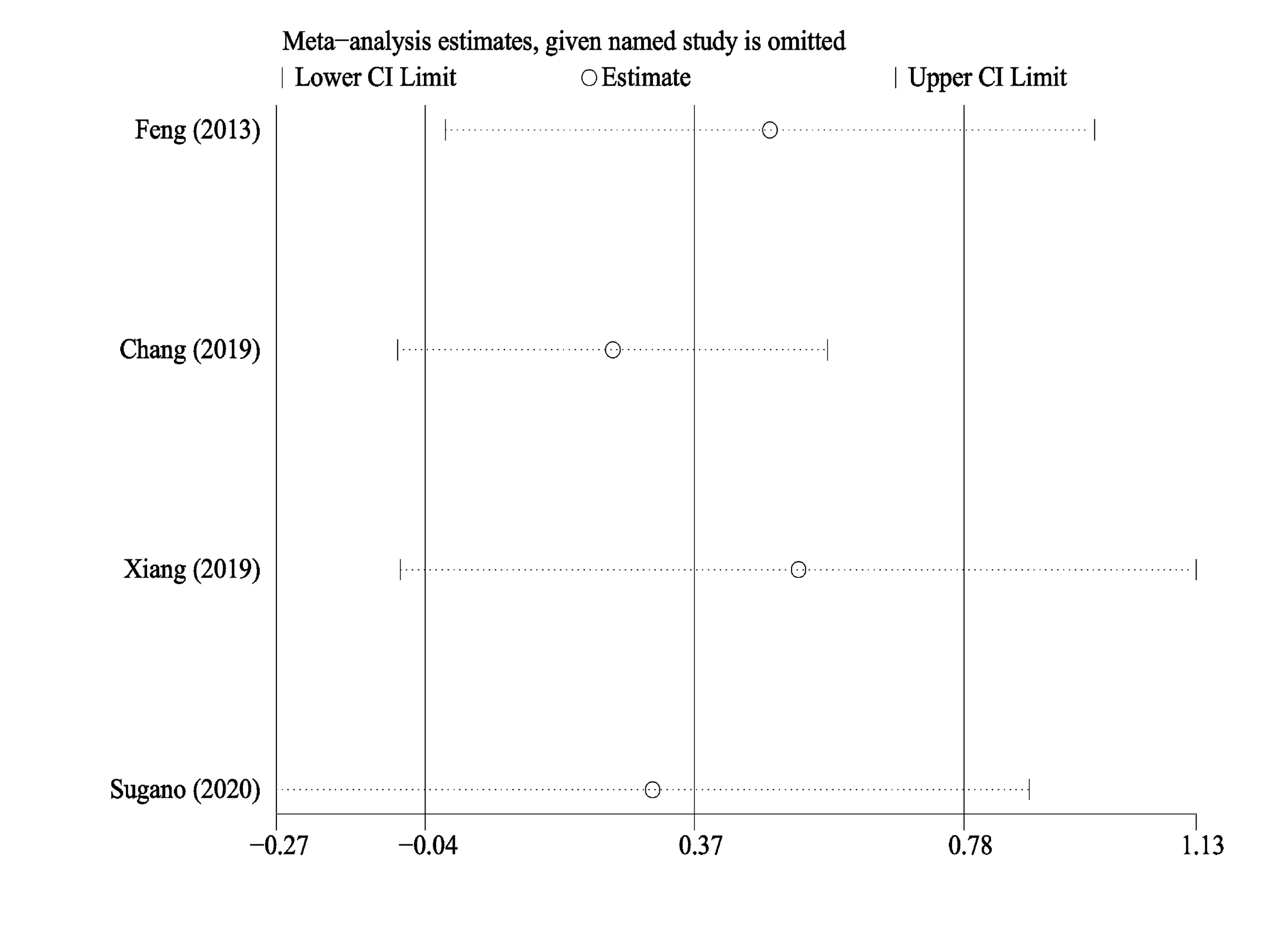

Supplement: S3 Fig — For relationship between SUA by categories (the lowest SUA category vs the median) and all-cause mortality in PD patients before recalculated the HRs and 95% CIs. HR, hazard ratio; CI, confidence interval. (TIF) [file pone.0264340.s004.tif]

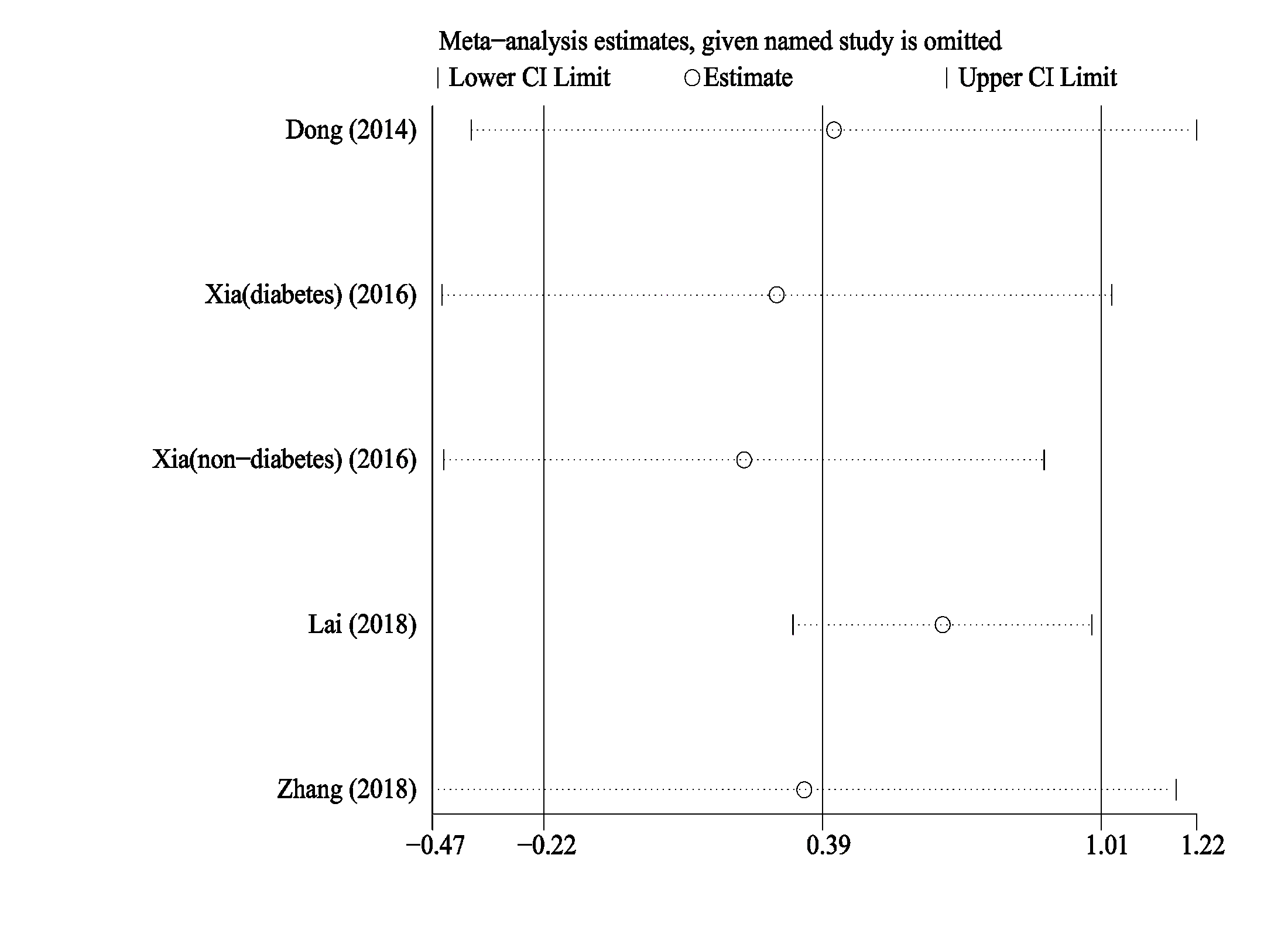

Supplement: S4 Fig — For relationship between SUA by categories (the highest SUA category vs the lowest) and cardiovascular mortality in PD patients before recalculated the HRs and 95% CIs. HR, hazard ratio; CI, confidence interval. (TIF) [file pone.0264340.s005.tif]

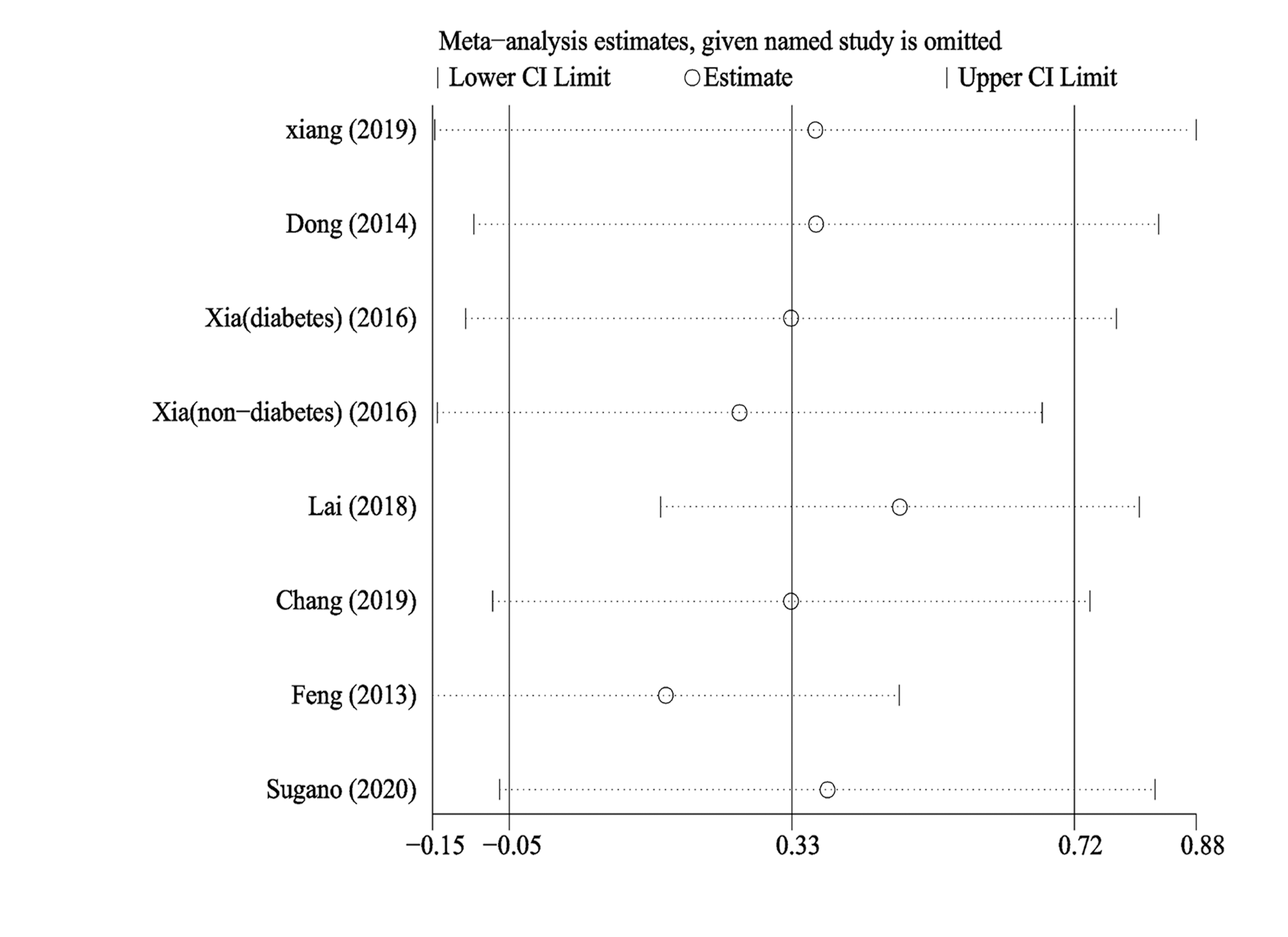

Supplement: S5 Fig — For relationship between SUA by categories (the highest SUA category vs the lowest) and all-cause mortality in PD patients after recalculated the HRs and 95% CIs. HR, hazard ratio; CI, confidence interval. (TIF) [file pone.0264340.s006.tif]

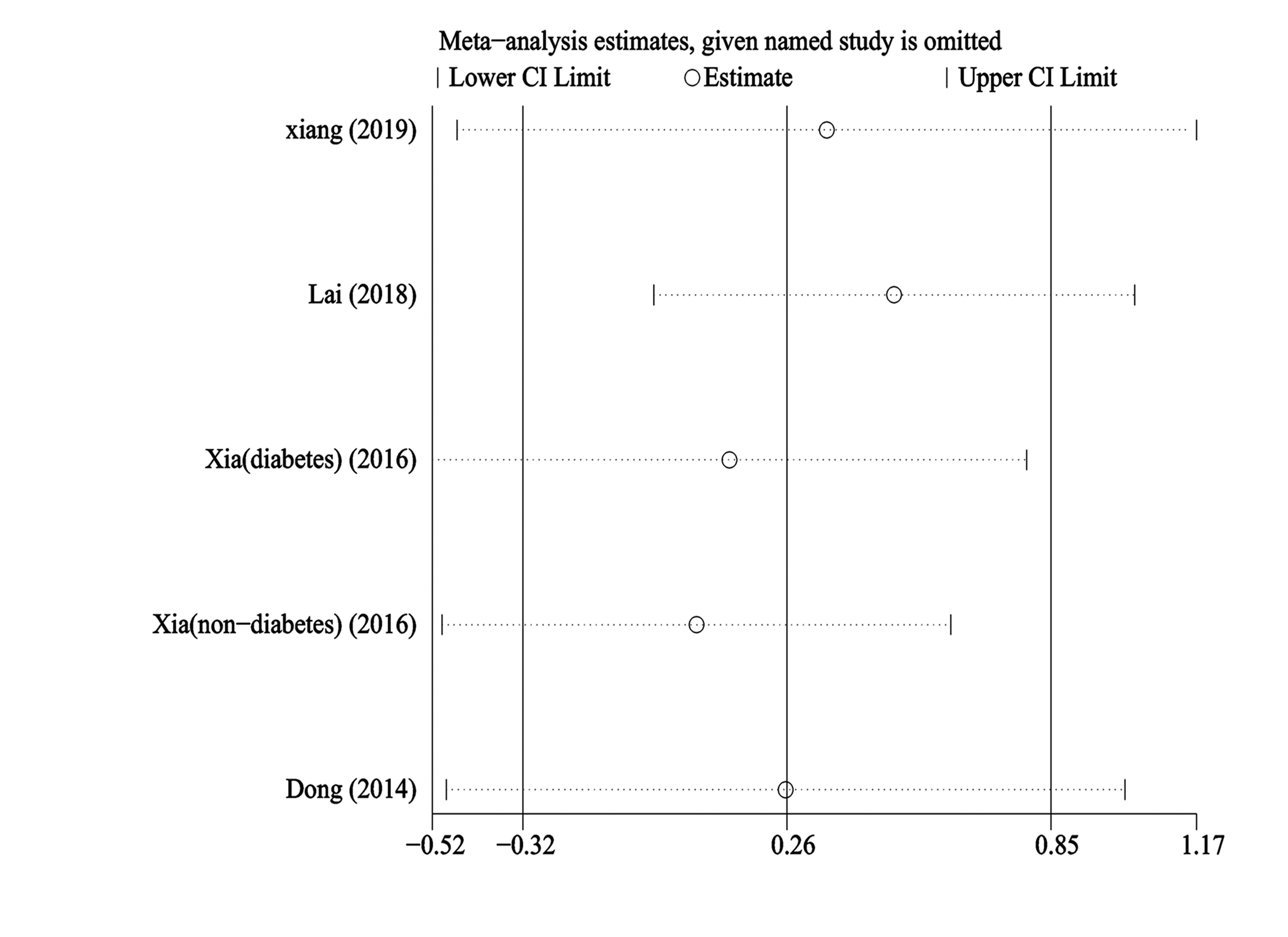

Supplement: S6 Fig — For relationship between SUA by categories (the highest SUA category vs the lowest) and cardiovascular mortality in PD patients after recalculated the HRs and 95% CIs. HR, hazard ratio; CI, confidence interval. (TIF) [file pone.0264340.s007.tif]
