# Supplementary material for: Serum uric acid level and all-cause and cardiovascular mortality in peritoneal dialysis patients: A systematic review and dose-response meta-analysis of cohort studies
Source: PLoS One. 2022 Feb 22;17(2):e0264340. doi: 10.1371/journal.pone.0264340 (PMC8863225; doi:10.1371/journal.pone.0264340)
Supplement: S1 Table — (DOCX) [file pone.0264340.s008.docx]

**S1 Table. Searching strategies for electronic databases.**

| **Databases** | **Searching strategies** | **Results (n)** |
| --- | --- | --- |
| **PubMed** | #1. ("Peritoneal Dialysis"[Mesh]) OR (((Dialyses, Peritoneal) OR (Dialysis, Peritoneal)) OR (Peritoneal Dialyses))  #2. ("Uric Acid"[Mesh]) OR (((((((((((((((Acid, Uric) OR (2,6,8-Trihydroxypurine)) OR (Trioxopurine)) OR (Potassium Urate)) OR (Urate, Potassium)) OR (Acid Urate, Ammonium)) OR (Urate, Ammonium Acid)) OR (Sodium Acid Urate)) OR (Urate, Monosodium)) OR (Urate, Sodium)) OR (Acid Urate, Sodium)) OR (Urate, Sodium Acid)) OR (Sodium Urate Monohydrate)) OR (Monohydrate, Sodium Urate)) OR (Urate Monohydrate))  #3. #1 AND #2 | 231 |
| **Embase** | #1. ‘Peritoneal dialysis’/ exp  #2. ‘Uric acid’/ exp  #3. ‘Dialyses, Peritoneal’: ab, ti  #4. ‘Dialysis, Peritoneal’: ab, ti  #5. ‘Peritoneal Dialyses’: ab, ti  #6. #1 OR #3 OR #4 OR #5  #7. ‘Acid, Uric’: ab, ti  #8. ‘Trioxopurine’: ab, ti  #9. ‘Potassium Urate’: ab, ti  #10. ‘Urate, Potassium’: ab, ti  #11. ‘Acid Urate, Ammonium’: ab, ti  #12. ‘Urate, Ammonium Acid’: ab, ti  #13. ‘Sodium Acid Urate’: ab, ti  #14. ‘Urate, Monosodium’: ab, ti  #15. ‘Urate, Sodium’: ab, ti  #16. ‘Acid Urate, Sodium’: ab, ti  #17. ‘Urate, Sodium Acid’: ab, ti  #18. ‘Monohydrate, Sodium Urate’: ab, ti  #19. ‘Urate Monohydrate’: ab, ti  #20. ‘Sodium Urate Monohydrate’: ab, ti  #21. #2 OR #7 OR #8 OR #9 OR #10 OR #11 OR #12 OR #13 OR #14 OR #15 OR #16 OR #17 OR #18 OR #19 OR #20  #22. #6 AND #21 | 340 |
| **Web of Science** | #1. TS=(Peritoneal dialysis OR Dialyses, Peritoneal OR Dialysis, Peritoneal OR Dialysis, Peritoneal)  #2. TS=(Uric acid OR Acid, Uric OR 2,6,8-Trihydroxypurine OR Trioxopurine OR Potassium Urate OR Urate, Potassium OR Urate, Potassium OR Acid Urate, Ammonium OR Urate, Ammonium Acid OR Sodium Acid Urate OR Urate, Monosodium OR Urate, Sodium OR Acid Urate, Sodium OR Urate, Sodium Acid OR Urate, Sodium Acid OR Sodium Urate Monohydrate OR Monohydrate, Sodium Urate OR Urate Monohydrate)  #3. #1 AND #2 | 373 |
| **The Cochrane library** | #1. MESH descriptor: [Peritoneal dialysis] explode all trees  #2. MESH descriptor: [Uric acid] explode all trees  #3. (Dialyses, Peritoneal): ti, ab, kw  #4. (Dialysis, Peritoneal): ti, ab, kw  #5. (Peritoneal Dialyses): ti, ab, kw  #6. #1 OR #3 OR #4 OR #5  #7. (Acid, Uric): ti, ab, kw  #8. (Trioxopurine): ti, ab, kw  #9. (Potassium Urate): ti, ab, kw  #10. (Urate, Potassium): ti, ab, kw  #11. (Acid Urate, Ammonium): ti, ab, kw  #12. (Urate, Ammonium Acid): ti, ab, kw  #13. (Sodium Acid Urate): ti, ab, kw  #14. (Urate, Monosodium): ti, ab, kw  #15. (Urate, Sodium): ti, ab, kw  #16. (Acid Urate, Sodium): ti, ab, kw  #17. (Urate, Sodium Acid): ti, ab, kw  #18. (Monohydrate, Sodium Urate): ti, ab, kw  #19. (Urate Monohydrate): ti, ab, kw  #20. (Sodium Urate Monohydrate): ti, ab, kw  #21. #2 OR #7 OR #8 OR #9 OR #10 OR #11 OR #12 OR #13 OR #14 OR #15 OR #16 OR #17 OR #18 OR #19 OR #20  #22. #6 AND #21 | 27 |
