# Supplementary material for: Serum uric acid level and all-cause and cardiovascular mortality in peritoneal dialysis patients: A systematic review and dose-response meta-analysis of cohort studies
Source: PLoS One. 2022 Feb 22;17(2):e0264340. doi: 10.1371/journal.pone.0264340 (PMC8863225; doi:10.1371/journal.pone.0264340)
Supplement: S2 Table — RCS, retrospective cohort study; PCS, prospective cohort study. (DOCX) [file pone.0264340.s009.docx]

**S2 Table. Quality assessment of the included studies utilizing the Newcastle-Ottawa Scale.**

| **Study ID** | **Selection** | | | | **Comparability** | **Outcome** | | | **Total score** |
| --- | --- | --- | --- | --- | --- | --- | --- | --- | --- |
|  | **Representativeness of exposed group** | **Representativeness of non-exposed group** | **Ascertainment of exposure** | **Demonstration that outcome was not present at start of study** | **Comparability of groups on the basis of design or analysis** | **Assessment of outcome** | **Follow up long enough for outcomes to occur** | **Adequacy of follow-up of groups** |  |
| Coelho, 2020 | ☆ | ☆ | ☆ | ☆ | ☆ | ☆ | ☆ | **-** | 7 |
| Sugano, 2020 | ☆ | ☆ | ☆ | ☆ | ☆ | ☆ | ☆ | ☆ | 8 |
| Xiao, 2020 | ☆ | ☆ | ☆ | ☆ | ☆ | ☆ | ☆ | ☆ | 8 |
| Xiang, 2019 | ☆ | ☆ | ☆ | **-** | ☆ | ☆ | ☆ | ☆ | 7 |
| Chang, 2019 | ☆ | ☆ | ☆ | **-** | ☆☆ | ☆ | ☆ | ☆ | 8 |
| Chang, 2019 | ☆ | ☆ | ☆ | **-** | ☆☆ | ☆ | **-** | ☆ | 7 |
| Lai, 2018 | ☆ | ☆ | ☆ | **-** | ☆☆ | ☆ | ☆ | ☆ | 8 |
| Zhang, 2018 | ☆ | ☆ | ☆ | **-** | ☆☆ | ☆ | ☆ | ☆ | 8 |
| Hsieh, 2017 | ☆ | ☆ | ☆ | **-** | ☆ | ☆ | ☆ | ☆ | 7 |
| Xia, 2016 | ☆ | ☆ | ☆ | **-** | ☆☆ | ☆ | ☆ | ☆ | 8 |
| Dong, 2014 | ☆ | ☆ | ☆ | ☆ | ☆☆ | ☆ | ☆ | ☆ | 9 |
| Xia, 2014 | ☆ | ☆ | ☆ | ☆ | ☆ | ☆ | ☆ | ☆ | 8 |
| Feng, 2013 | ☆ | ☆ | ☆ | **-** | ☆☆ | ☆ | ☆ | ☆ | 8 |
