# Supplementary material for: Serum uric acid level and all-cause and cardiovascular mortality in peritoneal dialysis patients: A systematic review and dose-response meta-analysis of cohort studies
Source: PLoS One. 2022 Feb 22;17(2):e0264340. doi: 10.1371/journal.pone.0264340 (PMC8863225; doi:10.1371/journal.pone.0264340)
Supplement: Attachment — Submitted filename: Response to Reviewers.docx [file pone.0264340.s010.docx]

Dear reviewers and editors,

Thank you for your letter and for the reviewers’ comments concerning our manuscript entitled “Serum uric acid level and all-cause and CVD mortality in peritoneal dialysis patients: A systematic review and meta-analysis of cohort studies” (PONE-D-21-22383). Those comments were valuable and very helpful for revising and improving our paper. We have studied comments carefully and have made corrections which we hope meet with approval. Revised portions are marked in red. The main corrections in the paper and the responses to the reviewer’s comments are as follows:

**Response to Academic Editor:**

To explore the source of heterogeneity among studies, subgroup analyses were conducted according to study design (prospective or retrospective cohort study), number of center (multi-center or single center), publication years (2013-2016 or 2017-2020), sample size (< 900 or > 900), follow-up duration (< 30 months or > 30 months), adjustment for gender and adjustment for diabetes status. And we also carefully revised the content and grammar of our article, hoping to improve the article.

**Response to additional Editor:**

To question 1: We provided details of NOS quality assessment for each study in the S2 Table.

To question 2: Sensitivity analysis, in which 1 study was removed at a time, was performed to evaluate the stability of the results. We added several sensitivity analyses, and the results are shown in S1-S6 Figs.

To question 3: It is really true as editor suggested that visual inspection of the funnel plot for publication bias is used when the number of the studies is more than or equal to 10 for a particular outcome. Therefore, we deleted the funnel plots and recorded the *P* values of egger’s test in the manuscript.

**Response to Reviewer:**

To question 1: We re-framed the objective of this study as follows: hence, the objective of this study is to determine the association between SUA and all-cause and CVD mortality with a detailed analysis of eligible literature.

To question 2:

(1) We have re-written this part according to the Reviewer’s suggestion: all non-English studies were translated by software first, and then the researchers checked whether they could be included.

(2) In addition to a comprehensive search, we considered the reference lists of all eligible articles in order to find additional relevant articles. I regret that unpublished data was not considered.

(3) We are very sorry that we did not search the Cochrane Library database before. Therefore, we limited the date filter to April 7, 2021, and finally retrieved 27 literatures (See S1 Table for searching strategies).

(4) We are very sorry that we did not register for this meta-analysis, but we conducted this systematic review and meta-analysis in strict accordance with the Preferred Reporting Items for Systematic Reviews and Meta-Analyses (PRISMA) statement.

To question 3: We have adjusted the resolution and size of the figures, hoping to meet the requirements of the journal.

To question 4: After consideration, we have included a conference abstract by Coelho et al [1] in Portugal, but the pooled results did not change. Nevertheless, almost all the studies included in the meta-analysis were conducted in Asian countries, which greatly limits the applicability of the results to the global population.

We are trying to upload original data, figures, tables and other information to the public database, but this will take some time. Once we get DOI, we will contact you immediately. In addition, I'm sorry that the command input in Stata software was wrong before, so we re-analyzed all the data. Before conducting meta-analysis, HR and 95%CIs need to be transformed as follows:

gen lnHR=ln(HR)

gen lnLL=ln(Lower CI)

gen lnUL=ln(Higher CI)

gen selogrr=(lnUL-lnLL)/(3.92)

Once again, thank you very much for your comments and suggestions. Looking forward to hearing from you. Best wishes.

Corresponding author:

Name: Quanjun Lyu

E-mail: lvquanjun666@163.com

Yours sincerely,

Quanjun Lyu

20211208

References

1.	Coelho ID, Rodriguez C, Rodriguez-Carmona A, Fontan MP. Serum uric acid, mortality, and decline of residual kidney function in patients undergoing chronic peritoneal dialysis. Journal of the American Society of Nephrology. 2020;31:420.
